# Intermittent water stress favors microbial traits that better help wheat under drought

**DOI:** 10.1093/ismeco/ycae074

**Published:** 2024-05-20

**Authors:** Ruth Lydia Schmidt, Hamed Azarbad, Luke Bainard, Julien Tremblay, Etienne Yergeau

**Affiliations:** Centre Armand-Frappier Santé Biotechnologie, Institut national de la recherche scientifique, Laval, QC, H7V 1B7, Canada; Department of Biology, Evolutionary Ecology of Plants, Philipps-University Marburg, Karl-von-Frisch-Strasse 8, 35043 Marburg, Germany; Agassiz Research and Development Centre, Agriculture and Agri-Food Canada, 6947 #7 Highway, Agassiz, BC, V0M 1A2, Canada; Centre Armand-Frappier Santé Biotechnologie, Institut national de la recherche scientifique, Laval, QC, H7V 1B7, Canada; Centre Armand-Frappier Santé Biotechnologie, Institut national de la recherche scientifique, Laval, QC, H7V 1B7, Canada

**Keywords:** wheat, drought, microbial communities, metagenomics, plant-microbe interactions, rhizosphere

## Abstract

Microorganisms can improve plant resistance to drought through various mechanisms, such as the production of plant hormones, osmolytes, antioxidants, and exopolysaccharides. It is, however, unclear how previous exposure to water stress affects the functional capacity of the soil microbial community to help plants resist drought. We compared two soils that had either a continuous or intermittent water stress history (WSH) for almost 40 years. We grew wheat in these soils and subjected it to water stress, after which we collected the rhizosphere soil and shotgun sequenced its metagenome. Wheat growing in soil with an intermittent WSH maintained a higher biomass when subjected to water stress. Genes related to indole-acetic acid and osmolyte production were more abundant in the metagenome of the soil with an intermittent WSH as compared to the soil with a continuous WSH. We suggest that an intermittent WSH selects traits beneficial for life under water stress.

## Introduction

Plant- and soil-associated microorganisms play a pivotal role in mitigating yield losses due to drought. These microorganisms have evolved diverse mechanisms to resist or avoid drought, which can also be beneficial to plants [1]. Unlike avoidance mechanisms like dormancy or sporulation, resistance mechanisms allow microorganisms to remain active and support plants during drought. Key resistance mechanisms include osmolyte production to retain water within cells [2], extracellular polymeric substances (EPS) production to enhance soil water retention [3], and the production of various enzymes to detoxify reactive oxygen species (ROS) generated under stress [4]. These mechanisms not only help microorganisms but also have positive effects on plants during drought. For example, microbially produced osmolytes can be transferred to plant tissues [5, 6], and microbially produced EPS near plant roots can enhance water-holding capacity and plant drought tolerance [7, 8]. Microorganisms also modulate the plant’s hormonal response to stress. The microbial 1-aminocyclopropane-1-carboxylate (ACC) deaminase degrades the precursor of the stress hormone ethylene [9]. Furthermore, the production of auxins and cytokinins by microorganisms can promote plant growth and resistance to stress [10, 11].

Although interfering with the regular plant stress response might offer short-term benefits, it could have long-term consequences, especially since larger plants are more susceptible to drought [12]. The overall beneficial impact of microbial activities on crops during drought depends on factors, such as the prevalence, abundance, diversity, and expression of traits, which, in turn, are influenced by various biotic and abiotic factors, including water availability.

Microbial communities respond to actual water availability in their environment, either through resistance or avoidance mechanisms. This selective pressure, when sustained or repeated over time, can lead to lasting shifts in microbial community composition and activities. The frequency of the stress event is also critical, as intermittent stress selects for generalists adapted to both stressful and normal conditions, whereas continuous stress favors microorganisms specialized for life under stressful conditions [13, 14]. For example, the microbial communities in two adjacent dryland wheat field soils subjected to intermittent or continuous water stress over nearly 40 years not only differed in composition but also responded differently to water stress [15, 16]. When wheat grew in soil with a continuous history of water stress, root biomass decreased more sharply when subjected to subsequent water stress compared to roots in soil with an intermittent water stress history (WSH) [17]. Additionally, microbial communities extracted from soil with an intermittent WSH reduced catalase activity in the leaves (an indicator of lower stress levels) when inoculated onto wheat growing in different soils and subjected to water stress [18]. Since these studies relied on amplicon sequencing, it remains unclear how the differences observed in microbial communities of soils historically subjected to different frequencies of stressful events translate to variations in microbial traits and their impact on plant stress resistance.

In this study, we used shotgun sequencing to analyze the rhizosphere metagenome of wheat plants from the soils of Azarbad *et al.* [16, 17], which had continuous or intermittent water stress histories. Our hypothesis was that intermittent water stress, due to its variable selection pressure, would favor a greater prevalence and diversity of functional traits related to surviving at low water availability, contrasting with the constant WSH. This increased diversity and prevalence of microbial traits is expected to enhance plants’ resistance to water stress events.

## Material and methods

### Soil material

The soil utilized in our pot study was sourced from two adjacent experimental fields (Swift Current, Saskatchewan, 50°17′ N; 107°41′ W) that had been subjected to distinct irrigation since 1981. Both fields followed wheat-fallow 2-year rotations, but one field was irrigated during the wheat phase of the rotation while the other remained unirrigated. Since the fields are in the semi-arid region of Saskatchewan, this difference in irrigation resulted in continuous and intermittent water stress conditions. Some of the physico-chemical properties of the soil in the two fields differed, but the magnitude of these differences was small (see Table S1 of [16]). Approximately 80 kg from the 0–30 cm layer of each of the fields was collected in the spring of 2016 and shipped to Laval, Québec, to set up a pot experiment.

### Experimental design and sampling

We sieved the soil at 2 mm and distributed 1 kg of it in pots. The pots were seeded with eight seeds of *Triticum aestivum* cv. AC Nass, a drought-sensitive bread wheat cultivar, and arranged in five experimental blocks in a growth chamber at 23°C with a 16:8-h light-dark photoperiod. For the first 4 weeks, soil water content was kept at 50% of the soil water holding capacity (SWHC) for all pots, after which it was either kept at 50% SWHC or brought down to 5% SWHC for another 4 weeks. This resulted in a total of four treatments (2 soil water stress histories × 2 soil water contents) with five replicates each, for a total of 20 samples. It should be noted that this is a subset of the pot study conducted by Azarbad *et al.* [16, 17]. These treatments were chosen among the entire experimental design based on previous studies that showed large contrasts in microbial communities [15–17]. At the end of the experiment, plants were uprooted and vigorously shaken, and the rhizosphere soil that remained attached to the roots was harvested, flash-frozen in liquid nitrogen, and kept at −80°C until DNA extraction. Plant biomass was divided into root and shoot portions and then weighed to obtain fresh weight and dried at 75°C for 48 hr to obtain dry weight (DW). The last emerging leaf was sampled, weighed (W), and hydrated to full turgidity in water for 2 hr. The leaves were then surface dried and weighed to obtain their turgid weight (TW). The leaves were finally oven-dried at 75°C for 48 hr to determine the DW. The leaf relative water content (RWC) [19], moisture content, and dry matter content were calculated as follows:


$$ \mathrm{RWC}=\frac{\mathrm{W}-\mathrm{DW}}{\mathrm{TW}-\mathrm{DW}}\times 100 $$

$$ \mathrm{Leaf}\ \mathrm{moisture}=\frac{\mathrm{W}-\mathrm{DW}}{\mathrm{DW}} $$

$$ \mathrm{Leaf}\ \mathrm{dry}\ \mathrm{matter}\ \mathrm{content}=\frac{\mathrm{TW}}{\mathrm{DW}}. $$

### DNA extraction and sequencing

The DNA was extracted using a DNeasy PowerSoil kit (Qiagen) and sent for metagenomic sequencing at the Centre d’expertise et de services Génome Québec located in Montréal, Québec. The sequencing procedure performed using an Illumina HiSeq 4000 (PE150) yielded a total of 699 061 058 reads, with an average of 34 953 053 reads per sample, resulting in a total of 105 Gbp, or an average of 5.2 Gbp per sample. The raw data has been deposited under BioProject accession PRJNA1040208.

### Bioinformatics

The sequencing reads were processed using our established metagenomic pipeline (ShotgunMG v1.3.2), as previously described [20, 21]. Briefly, sequencing adapters were removed from each read, and bases at the end of reads having a quality score <30 were cut off (Trimmomatic v0.32) [22] and scanned for sequencing adapters contaminants reads using DUK (http://duk.sourceforge.net/) to generate quality-controlled (QC) reads. QC-passed reads from each sample were co-assembled using Megahit v1.1.2 [23] with iterative kmer sizes of 31, 41, 51, 61, 71, 81, and 91 bases. Gene prediction was performed by calling genes on each assembled contig using Prodigal v2.6.2 [24]. Genes were annotated following the JGI’s guidelines [25], including the assignment of KEGG orthologs (KO). QC-passed reads were mapped (BWA mem v0.7.15) (unpublished, http://bio-bwa.sourceforge.net) against contigs to assess the quality of metagenome assembly and to obtain contig abundance profiles. Alignment files in bam format were sorted by read coordinates using samtools v1.2 [26], and only properly aligned read pairs were kept for downstream steps. Each bam file was analyzed for coverage of called genes and contigs using bedtools (v2.17.0) [27] using a custom bed file representing gene coordinates on each contig. Only paired reads that overlapped their contig or gene were considered for gene counts. Coverage profiles of each sample were merged to generate an abundance matrix (rows = contig, columns = samples), for which we calculated corresponding CPMs (counts per million—normalized using the TMM method) (edgeR v3.10.2) [28]. Each contig was blasted (BLASTn v2.6.0+) against NCBI’s NT database (version downloaded from NCBI’s server on 9 January 2019), and the best hit’s taxonomic identifier was used to assign a taxonomic lineage to the contig. Taxonomic summaries were performed using MicrobiomeUtils v0.9 (github.com/microbiomeutils). Subsequently, reads were mapped onto the contigs to derive abundance profiles, which were used as input to generate metagenome-assembled genomes (MAGs) (maxbin2), whose quality was checked using checkM [29]. We only analyzed MAGs that had a completion over 50% and <10% contamination, corresponding to the “medium-quality” threshold for MAGs [30].

**Table 1:** Anova table for plant root and shoot biomass, and leaf moisture content, relative water content, and dry matter content for wheat subjected to water stress and growing in two soils with contrasting soil water stress history.

		Root fresh biomass (g)		Shoot fresh biomass (g)		Root dry biomass (g)		Shoot dry biomass (g)		Leaf moisture		Leaf relative water content (%)		Leaf dry mater content (mg/g)	
Intermittent WSH	HWC	0.37	a	1.36	a	0.52	a	0.20	a	8.25	a	97.1	a	0.11	a
	LWC	0.11	b	0.24	b	0.008	b	0.06	b	3.67	b	63.4	b	0.15	bc
Continuous WSH	HWC	0.73	c	1.26	a	0.54	a	0.21	a	7.95	a	97.1	a	0.12	ac
	LWC	0.07	b	0.16	c	0.05	b	0.05	b	2.86	b	59.4	b	0.18	b
Soil type	F	1.13		6.22		0.11		0.49		2.02		0.20		6.13	
	P	0.31		0.028	^*^	0.75		0.50		0.18		0.67		0.029	^*^
% SWHC	F	229.12		397.47		40.39		228.16		153.11		62.67		34.23	
	P	3.5 × 10^−9^	^*^^*^^*^	1.45 × 10^−10^	^*^^*^^*^	3.63 × 10^−5^	^*^^*^^*^	3.59 × 10^−9^	^*^^*^^*^	3.43 × 10^−8^	^*^^*^^*^	4.19 × 10^−6^	^*^^*^^*^	7.83 × 10^−5^	^*^^*^^*^
Soil:SWHC	F	21.72		3.98		1.10		2.12		0.42		0.19		0.81	
	P	0.00055	^*^^*^^*^	0.069	.	0.32		0.17		0.53		0.67		0.39	

^*^^*^^*^: *P* < .001; ^*^^*^: .001 < *P* < 0.01; ^*^: .01 < *P* < .05; .: .05 < *P* < .10.

Different letters indicate significant differences at *P* < .05 in Tukey HSD tests.

### Functional traits

For the functional trait analyses, we searched our gene annotation table for entries related to the functions of interest using their KO entries. For the ACC deaminase, we used the only KO available for this function: K01505. For IAA production, we used KOs of enzymes that led directly to IAA in the tryptophan metabolism map (map00380): K01501, K01426, K21801, K11816, K11817, and K00128. For osmolyte production, we used the KO list presented in Supplementary Table 2 of McParland *et al.* [31]. For EPS biosynthesis, we used the 73 KOs associated with the KEGG pathway ko00543 (exopolysaccharide biosynthesis). For cytokinin, we used the 10 KOs associated with the KEGG pathway ko00908 (Zeatin biosynthesis). For antioxidants, we searched for KOs with the terms “superoxide dismutase” (5 entries), “glutathione peroxidase” (4 entries), “catalase” (4 entries), or “cytochrome oxidase” (58 entries). The complete list of KO entries used is available in the “05-Traits.R” script in our GitHub repository (see below).

### Statistics

All R code used for data manipulation, statistical analyses, and figure generation can be found on our GitHub repository (https://github.com/le-labo-yergeau/MG_Growth_Room). The data employed in the R scripts have been deposited on the Zenodo platform (https://zenodo.org/doi/10.5281/zenodo.10140592). Briefly, we used ANOVA and *post-hoc* Tukey HSD to test the effect of soil water content and WSH on root and shoot fresh and DWs and shoot water content. We used principal coordinate analyses (PCoA) based on Bray–Curtis dissimilarity calculated from the entire gene abundance table to visualize the differences between the treatments and tested these differences using permutational multivariate ANOVA (permANOVA). For the different functional traits listed above, we compared their total abundance (sum of all genes related to a trait) across treatments using ANOVA and post-hoc Tukey HSD tests, whereas we compared their “community composition” using permANOVA on a subset of the gene abundance table. Finally, ANOVAs with Bonferroni correction for multiple testing (*P* < .05/68) were used to identify MAGs that were affected by the treatments.

## Results

### Plant traits

**Figure 1:**
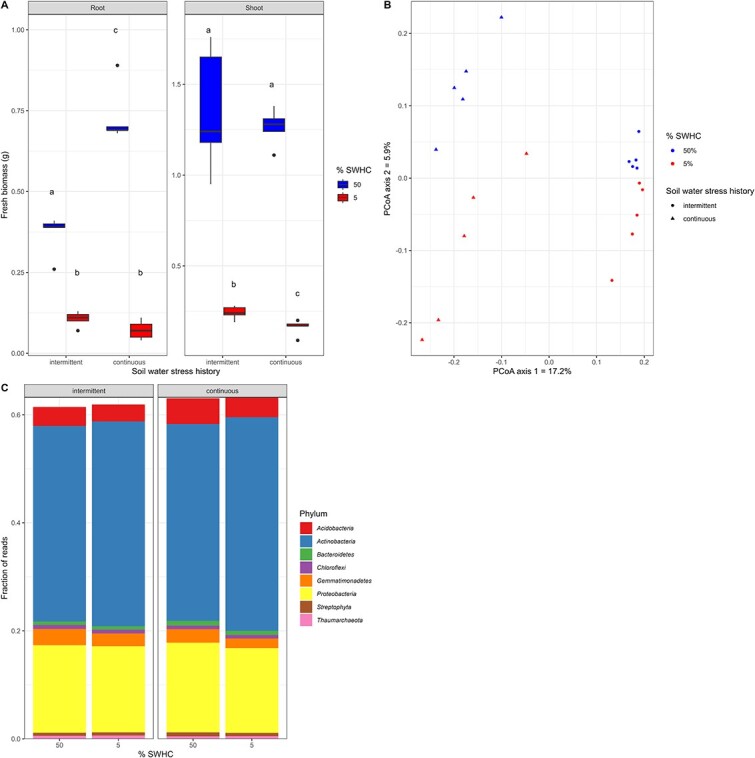
Plant and general microbial responses. (A) Root and shoot fresh biomass, (B) metagenomic community structure using a principal coordinate analysis of Bray–Curtis dissimilarities, and (C) metagenomic community composition for rhizosphere samples taken from wheat growing in soil with an intermittent or continuous water stress history and subjected to low (5% SWHC) or high (50% SWHC) water availability. Different letters in (A) indicate significant differences at *P* < .05. The whiskers extend from the hinge to the largest (top) or smallest (bottom) value no further than 1.5 × the IQR (inter-quartile range) from the hinge.

In comparison to the high soil water content (50% SWHC) treatment (HWC), the low soil water content (5% SWHC) treatment (LWC) reduced plant fresh biomass by 83.8% (roots) to 84.6% (shoots), on average (*P* < .001, Table 1, Fig. 1A). For shoots, this reduction in fresh biomass can be attributed, at least in part, to a 59.7% reduction in leaf moisture and a 36.8% decrease in leaf RWC (*P* < .001, Table 1), but also to a 71.6% decrease in shoot dry biomass (*P* < .001, Table 1). Soil WSH also had an impact on shoot fresh biomass, with fresh biomass being reduced by 10.8% in the continuous WSH soil as compared to the intermittent WSH soil, which is most evident under LWC (*P* = .028, Table 1, Fig. 1A). Additionally, the fresh root biomass of HWC plants was 49.3% higher for plants growing in the soil with a continuous WSH compared to the plant growing in the soil with an intermittent WSH, but this was not significant under LWC (interaction term: *P* < .001, Table 1, Fig. 1A). Neither WSH nor the interaction term affected the leaf moisture or RWC (*P* > .05, Table 1), suggesting that their effect on leaf fresh biomass was not due to changes in water content. The leaf dry matter content was 31.5% higher in the LWC treatment as compared to the HWC treatment (*P* < .001, Table 1), and 14.7% higher in the continuous WSH soil as compared to the intermittent WSH soil (*P* < .05, Table 1).

**Table 2:** Permanova results for the composition of functional genes in the rhizosphere of wheat subjected to water stress and growing in two soils with contrasting soil water stress history.

		Soil type	%SWHC	Soil: %SWHC
All genes	F	6.98	1.45	1.14
	P	0.0001^*^^*^^*^	0.16	0.26
IAA	F	7.17	1.54	1.17
	P	0.0001^*^^*^^*^	0.13	0.26
ACC	F	8.11	1.57	1.30
	P	0.0001^*^^*^^*^	0.14	0.22
Osmolytes	F	7.76	1.44	1.14
	P	0.0001^*^^*^^*^	0.17	0.28
EPS	F	7.20	1.41	1.11
	P	0.0001^*^^*^^*^	0.17	0.28
Cytokinines	F	7.05	1.44	1.06
	P	0.0002^*^^*^^*^	0.16	0.33
ROS	F	7.76	1.44	1.14
	P	0.0001^*^^*^^*^	0.17	0.28

^*^^*^^*^: *P* < .001; ^*^^*^: .001 < *P* < .01; ^*^: .01 < *P* < .05; .: .05 < *P* < .10.

### Microbial community composition

The gene community structure (based on all genes) showed that rhizosphere samples from wheat growing in the same soil were more similar to each other than to the other soil (PCoA of Bray–Curtis dissimilarity: Fig. 1B and Permanova: Table 2, *F* = 6.98, *P* = .0001). The current soil water content also resulted in a separation of the wheat rhizosphere samples on the second axis of the ordination (Fig. 1B), but this trend was not significant in Permanova tests (*F* = 1.45, *P* = 0.16, Table 2). The community composition at the phylum level did not differ (Fig. 1C), suggesting that the two soils were taxonomically similar at that level. The annotated reads were mainly affiliated with the Actinobacteria and, to a lesser extent, the Proteobacteria (Fig. 1C).

### Microbial drought-related traits

**Figure 2:**
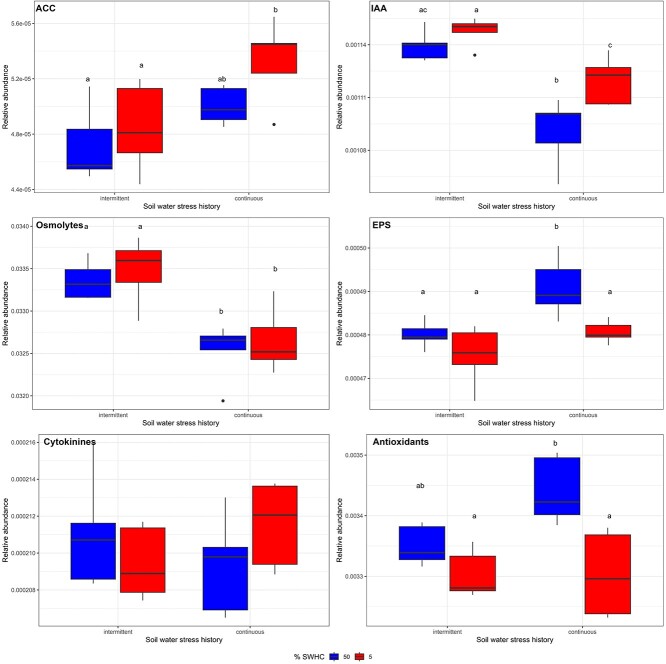
Relative abundance of genes encoding for drought-related functional traits. ACC deaminase, indole acetic acid, osmolytes, EPS, cytokinins, and antioxidants relative abundance for rhizosphere samples taken from wheat growing in soil with an intermittent or continuous water stress history and subjected to low (5% SWHC) or high (50% SWHC) water availability. Different letters indicate significant differences at *P* < .05. The whiskers extend from the hinge to the largest (top) or smallest (bottom) value no further than 1.5 × the IQR (inter-quartile range) from the hinge.

We compared the total abundance and composition of six genes/pathways that could be involved in microbial beneficial services to the plants under water stress: indoleacetic acid (IAA) synthesis, ACC deaminase synthesis, cytokinin metabolism, EPS synthesis, osmolyte production, and antioxidant synthesis. Like the patterns observed for all genes, the gene composition of the subgroups was only influenced by the WSH (*P* < .0001 for all, Table 2). However, when looking at the summed abundance of the genes in a subgroup, different patterns emerged (Fig. 2, Table 3). For instance, the relative abundance of IAA and EPS-related genes and of the ACC deaminase were influenced by both the WSH and the current soil water content; the relative abundance of osmolyte-related genes was only influenced by the WSH; the relative abundance of antioxidants was only influenced by the actual soil water content; and the relative abundance of cytokinins was not influenced by any of the experimental factors (Table 3). Even if there were similarities in the factors affecting these groups of genes, the patterns were not the same. The ACC-deaminase gene was 7% more abundant in the rhizosphere of wheat growing in soil with a continuous WSH, and it was also 5% more abundant in the LWC rhizospheres (Fig. 2). IAA-related genes were 3% more abundant in the soil with an intermittent WSH as compared to the soil with a continuous WSH (Fig. 2). Similarly, IAA genes were 1.6% more abundant in the LWC treatment as compared to the HWC treatment (Fig. 2). Osmolyte genes were 2% more abundant in the rhizosphere of plants growing in the soil with an intermittent WSH (Fig. 2). EPS production genes were 1.7% more abundant in the soil with continuous WSH and 1.6% more abundant in the HWC pots (Fig. 2). There was no significant difference for the cytokinins (Fig. 2). Antioxidant-related genes were 1.3% more abundant in the soils with continuous WSH and 2.7% more abundant in HWC soils (Fig. 2).

**Table 3:** Anova results for the relative abundance of functional genes in the rhizosphere of wheat subjected to water stress and growing in two soils with contrasting soil water stress history.

		Soil type	%SWHC	Soil: %SWHC
IAA	F	49.07	11.44	3.58
	P	1.42 × 10^−5^^*^^*^^*^	0.0055^*^^*^	0.083.
ACC	F	14.06	5.00	0.95
	P	0.0028^*^^*^	0.045^*^	0.35
Osmolytes	F	35.71	0.76	0.001
	P	6.45 × 10^−5^^*^^*^^*^	0.40	0.98
EPS	F	19.66	17.02	2.22
	P	0.00082^*^^*^^*^	0.0014^*^^*^	0.16
Cytokinins	F	0.025	0.084	3.02
	P	0.88	0.78	0.11
ROS	F	3.48	14.71	3.54
	P	0.087.	0.0024^*^^*^^*^	0.084.

^*^^*^^*^: *P* < .001; ^*^^*^: .001 < *P* < .01; ^*^: .01 < *P* < .05; .: .05 < *P* < .10.

### Metagenome-assembled genomes (MAGs)

**Figure 3:**
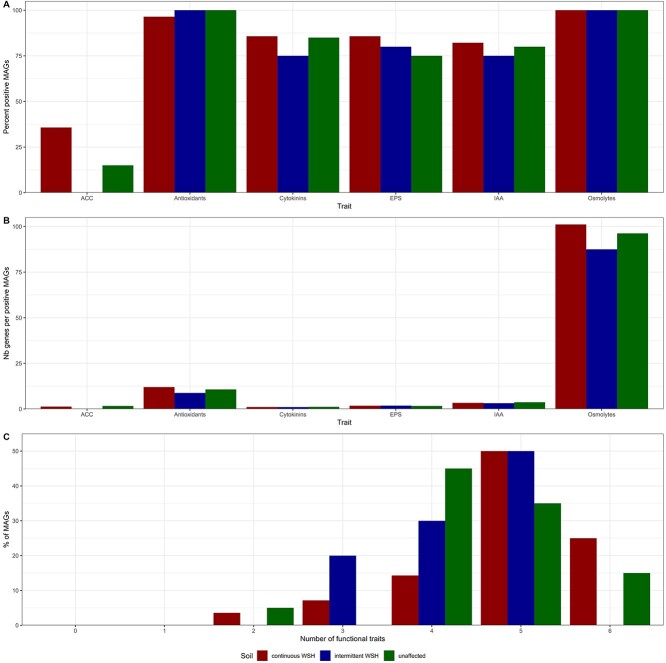
Functional traits in metagenome assembled genomes (MAGs). (A) Percentage of the significantly affected MAGs harboring at least one copy of the genes encoding for drought-related functional traits, (B) number of genes encoding for a drought-related functional trait per significantly affected MAG, for MAGs harboring at least one of such gene, (C) number of different traits harbored by the significantly affected MAGs. MAGs were assembled from shotgun metagenomic sequencing of rhizosphere samples taken from wheat growing in soil with an intermittent or continuous water stress history and subjected to low (5% SWHC) or high (50% SWHC) water availability. Since the values here are a list of MAGs that resulted from an ANOVA, statistical significance cannot be tested for. The third bar represents the unaffected MAGs. Continuous: *n* = 28, intermittent: *n* = 20, and unaffected: *n* = 20.

Among the 300 MAGs created, only 68 were medium- and high-quality MAGs (>50% completeness and <10% contamination), containing on average 8.05% of the total number of reads. Among these 68 MAGS, the relative abundances of 48 were affected by the soil WSH and only 5 by the actual soil water content (Bonferroni corrected *P* < .05/68). Among the 48 MAGs affected by soil history, 20 were enriched in the soil with intermittent WSH, and 28 were enriched in the soil with continuous WSH. The average completeness of the MAGs in the different categories differed, with 73.4%, 81.1%, and 71.8% for the intermittent WSH, the continuous WSH, and the unaffected MAGs, respectively. Although this makes it difficult to interpret the patterns observed between the MAG categories, some general trends emerged. For instance, except for the ACC deaminase, the drought-relevant functional traits were present in 75% or more of the MAGs (Fig. 3A), which resulted in 50% or more of the MAGs harboring five or more functional traits (Fig. 3C). Most of the functional traits were represented by an average number of genes a little over one, except for osmolytes, antioxidants, and IAA genes that were present on average 95, 11, and 3 times per MAG (Fig. 3B).

## Discussion

We compared the response of wheat and its microbiota to water stress when growing in soil with almost a 40-year history of contrasting water stress frequencies. In line with our hypothesis, we showed that the soil subjected to intermittent water stress better mitigated wheat fresh biomass loss in response to reduced soil water content because it was enriched with microorganisms with traits beneficial for plants under water stress.

Previous exposure to stress was shown to generate a microbiota that is better adapted when facing this stress again [32, 33], and this extends to beneficial services to plants. For instance, trees grown with a microbiota with a history of stress do better when facing the same stress [34], and *Brassica rapa* better resists water stress when grown in soil that was pre-exposed to water stress [35]. Here, we showed that the frequency of stress is also important. The soil microbiota with intermittent exposure to water stress better mitigated wheat biomass loss under low water content than the microbiota constantly exposed to water stress. Models showed that constant stressful conditions select for a microbial community dominated by a few specialists, increasing its sensitivity to environmental change and reducing its functional performance [13]. Intermittent water stress, in contrast, selected for microbial taxa that could grow at low and high water availability, i.e. generalists [13]. Experimentally subjecting a sulfidic stream microbiome to oxic and anoxic changes similarly selected for generalists active under both conditions [14]. Although this was difficult to assess with the varying completeness level and the relatively small number of MAGs created, the intermittent WSH treatment could have selected for generalists that harbored a wider diversity of drought-related functional traits. Harboring multiple traits is crucial for microorganisms to help plants during water stress. For instance, if a microorganism can mitigate plant response to stress, e.g. through interference with plant hormones, but cannot itself adapt to low soil water content, e.g. through the proficient production of osmolytes, then it will not be able to help plants during drought. Microbes combining many traits could resist low water content and, at the same time, promote plant growth. For instance, IAA and osmolyte production genes were relatively more abundant in the soil with an intermittent WSH, and 15 out of 20 (75%) of the intermittent WSH MAGs harbored both traits.

Osmolyte production was the most widely distributed trait in the microbial community, with all the MAGs harboring this trait and around 3% of all the reads being identified as osmolyte-related genes. Osmolyte production is one of the major mechanisms that microorganisms use to resist drought; it was estimated that 3%–6% of the total annual net primary production of a grassland ecosystem is used for that purpose by microbes during a drought event [36]. We previously identified bacterial and fungal osmolyte-related genes among the most differentially expressed genes in the wheat rhizosphere under reduced soil water content [21]. Here, osmolyte production was the trait that showed the largest response to WSH, being more abundant in the intermittent WSH soils. As bacteria can transfer osmolytes to plants [5, 6], it is possible that the intermittent WSH soil will result in better water retention in plant tissues, which could explain the patterns observed in aboveground and belowground plant fresh biomass. As mentioned above, osmolyte production alone might not be sufficient to mitigate water stress in plants, and a combination with other traits could be required.

Although soil water content dictated the plant’s biomass and leaf water content, it did not affect the microbial community structure. In contrast to the large influence of WSH, actual soil water content only influenced the relative abundance of five MAGs. Water stress normally decreases soil respiration [17] and microbial richness [37], increases the fungal–bacterial ratio [38], and shifts the microbial transcriptome [21]. We had reported for these soils that WSH constrained the response of microbial communities to actual water stress [15–17]. Alternatively, the timeframe of our experiment might have been too short for these changes to translate to shifts in functional and taxonomical composition of the microbial community. In all cases, this could lead to an uncoupling of the plant-microbe interactions as the two partners do not share the same environmental cues for their response to short-term stress. Eukaryotes—including plants—also use the microbiota as a cue for their development [39], which could abate this potential uncoupling. For example, independently of the actual drought conditions, a drought-adapted microbiota accelerated the flowering time of *Brasssica napa* as compared to a microbiota that was not adapted to drought [35].

Overall, we showed that a 40-year history of intermittent soil water stress selects a microbial community enriched in important traits for plant and microbial adaptation to low soil water availability. This community better mitigated the effects of water stress on wheat, with plants growing in their presence having higher fresh biomass under low soil water content. Microorganisms harboring many of these traits—generalists—could be a key group for microbially-mediated plant stress resistance. We now have a clearer target for our microbial community manipulation efforts, toward improving crops’ resistance to environmental stresses.

## Supplementary Material

SupplementaryTable1_ycae074

## Data Availability

The raw sequencing data has been deposited under BioProject accession PRJNA1040208. All R code used for data manipulation, statistical analyses, and figure generation can be found on GitHub (https://github.com/le-labo-yergeau/MG_Growth_Room). The data employed in the R scripts have been deposited on the Zenodo platform (https://zenodo.org/doi/10.5281/zenodo.10140592).
